# Greater than the sum of their parts: an overview of the AvrRps4 effector family

**DOI:** 10.3389/fpls.2024.1400659

**Published:** 2024-05-10

**Authors:** Katie N. Horton, Walter Gassmann

**Affiliations:** Division of Plant Science and Technology, Bond Life Sciences Center, and Interdisciplinary Plant Group, University of Missouri, Columbia, MO, United States

**Keywords:** effector, pathogen, Pseudomonas, Xanthomonas, virulence, HopK1, XopO, XopAK

## Abstract

Phytopathogenic microbes use secreted effector proteins to increase their virulence *in planta*. If these effectors or the results of their activity are detected by the plant cell, the plant will mount an immune response which applies evolutionary pressure by reducing growth and success of the pathogen. Bacterial effector proteins in the AvrRps4 family (AvrRps4, HopK1, and XopO) have commonly been used as tools to investigate plant immune components. At the same time, the *in planta* functions of this family of effectors have yet to be fully characterized. In this minireview we summarize current knowledge about the AvrRps4 effector family with emphasis on properties of the proteins themselves. We hypothesize that the HopK1 C-terminus and the AvrRps4 C-terminus, though unrelated in sequence and structure, are broadly related in functions that counteract plant defense responses.

## Introduction

Plants constantly interact with new challenges presented by their surroundings, and perhaps one of the most relevant interactions for humanity is that between a plant and a pest or pathogen attempting to benefit from the unique position of plants as primary nutrient sources. In the case of microbial biotrophic pathogens, the association between host and co-evolved pathogen is accompanied by intricate manipulation of the plant for the pathogen’s benefit (Jones and Dangl, 2006; Ebert and Fields, 2020). Usually manipulation involves secretion by the attacking pathogen of proteins called effectors into the plant cell which, broadly, attempt to improve the attacker’s virulence and ultimate ecological success by blocking plant recognition and resistance and/or by increasing availability of resources the attacker needs (McCann and Guttman, 2008; Feng and Zhou, 2012; Kazan and Lyons, 2014; Wang et al., 2022). Successful pathogens cause diseased crops, reduced yield and, in extreme cases, total crop loss. Identifying the *in planta* virulence targets, or the functions, of these effectors is the first step along a pathway that leads to developing crops with greater resistance to disease and eventually to greater food security. In this review, we take this first step by examining the well-known AvrRps4 effector family and reviewing published information that may provide insight into the *in planta* functions of each effector domain.

As the first isolated bacterial effector known to trigger resistance through a Toll/Interleukin-1 Receptor - Nucleotide Binding - Leucine Rich Repeat (TNL) resistance protein, AvrRps4 was used widely among plant-microbe researchers as a tool for the discovery and investigation of plant resistance components (Gassmann et al., 1999). Though AvrRps4 has been invaluable to the field of molecular plant-pathogen interactions, many facets of the effector’s virulence and avirulence targets *in planta* remain unknown.

**Figure 1:**
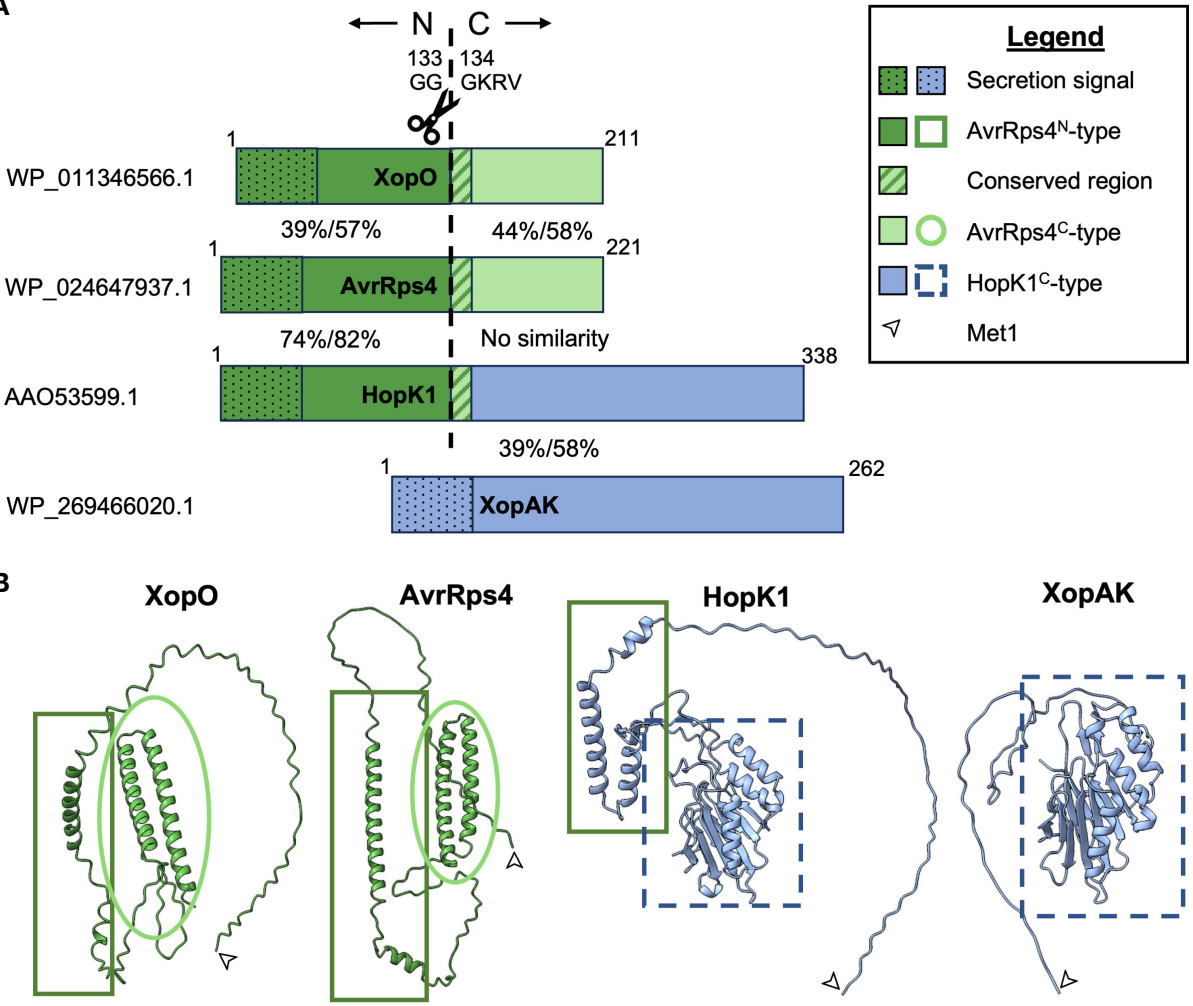
Visual representations of AvrRps4 effector family homology. **(A)** Proportional amino acid sequence homology indicated by color and pattern. AvrRps4^N^-type sequences are indicated in dark green, AvrRps4^C^-type sequences are indicated in light green, and HopK1^C^-type sequences are indicated in light blue. NCBI accession numbers for proteins used in this comparison are listed on the left. Percentages between the termini indicate % identity/% positives for each pair as predicted by NCBI’s blastp (Altschul et al., 1990). The ~50AA secretion signals (represented by dots) are included in this comparison but the 12AA central homologous regions (represented by stripes) are not. **(B)** Protein structural homology with Met1 indicated by an arrow. Structural predictions were made with ColabFold and visualized using ChimeraX (Pettersen et al., 2021; Mirdita et al., 2022).

The three members of the AvrRps4 effector family (AvrRps4, HopK1, and XopO) are characterized by high N-terminal homology to each other followed by a GGGKRVY motif. After secretion into a plant cell via the type III secretion system (T3SS), all members of the family are processed by an unknown protease(s) that cleaves the N-terminus from the C-terminus between GG and GKRVY. This occurs between G133 and G134 (G123 and 124 in XopO) and for unknown reasons requires an arginine at position 112 (R112) (R111 in XopO) (**Figure 1**) (Roden et al., 2004; Sohn et al., 2009; Li et al., 2014; Halane et al., 2018; Su et al., 2021; Nguyen et al., 2022). AvrRps4 and XopO possess homologous AvrRps4-type N- and C-termini (AvrRps4^N^ and AvrRps4^C^), but HopK1 only shares N-terminal homology with AvrRps4. HopK1 C-terminus (HopK1^C^) instead shares homology with XopAK (**Figure 1**) and RipBQ, a *Ralstonia* effector not discussed in this minireview due to limited space and literature (Sabbagh et al., 2019). Although XopAK is not a proper member of the AvrRps4 effector family due to lack of a GGGKRVY motif or any homology to AvrRps4^N^, it is present in multiple important bacterial crop pathogens, including those that cause banana Xanthomonas wilt, maize bacterial leaf streak, bacterial leaf streak of rice, and all currently known citrus canker strains (Moreira et al., 2010; Studholme et al., 2010; Jalan et al., 2011, 2013; Bart et al., 2012; Darrasse et al., 2013; Wichmann et al., 2013; Gagnevin et al., 2014; Aritua et al., 2015). We believe its inclusion will provide useful insights into the *in planta* function(s) of HopK1^C^.

This summary is intended to be used as a collection of knowledge on the topic and to be viewed as a description of possibilities to be explored further. We strive to consider the preponderance of evidence available rather than focusing on any singular result when we discuss these effectors below.

## AvrRps4

The founding member of the family, the *avrRps4* avirulence gene from *Pseudomonas syringae* pv. *pisi* (Psp) str. 151, was described in 1996 and was one of the first avirulence genes shown to be present in all tested strains of a pathovar (Hinsch and Staskawicz, 1996). In both Psp 151 and *P. syringae* pv. *phaseolicola* str. 1448A, *avrRps4* is located on a plasmid (Kim et al., 1998; Joardar et al., 2005).

After secretion and subsequent processing inside the plant cell, both AvrRps4 termini are largely nucleo-cytoplasmically localized (Bhattacharjee et al., 2011; Heidrich et al., 2011; Sohn et al., 2012; Li et al., 2014; Park et al., 2017; Halane et al., 2018). Interestingly, Li et al., 2014 also reported N-terminus-dependent localization of transgenically expressed AvrRps4 and HopK1 to the chloroplast. Independently, *in vitro* import assays showed localization of HopK1 in chloroplasts (De Torres Zabala et al., 2015). More recent work has shown that deliberate AvrRps4 mislocalization to the plasma membrane by replacing the N-terminus does not prevent processing, accumulation of the C-terminus in the nucleus, or contribution to resistance (Halane et al., 2018). AvrRps4^N^ (aa 1-136) in particular has been used with varied success in the past as part of the effector detector vector system (pEDV) for identification of candidate effectors from other bacteria (Kim et al., 2023), oomycetes (Sohn et al., 2007; Fabro et al., 2011; Kemen et al., 2011; Badel et al., 2013; Upadhyaya et al., 2014), fungi (Sharma et al., 2013; Zhang et al., 2020), insects (Navarro-Escalante et al., 2020; Peng et al., 2023), and nematodes (Shi et al., 2018b, 2018a). This, alongside the apparent similarities between N-terminal secretion signals of type III secreted effector proteins and chloroplast/mitochondrial transit peptides (Guttman et al., 2002) indicate that while a portion of the cellular pool of AvrRps4 may be targeted to the chloroplast for its virulence function, presence of AvrRps4^N^ is likely not the only factor leading to the observed chloroplast localization.

Further, the AvrRps4 N-terminus appears to have a greater role in virulence than exclusively as a signal peptide. Transgenic expression of AvrRps4^N^ alone in *Arabidopsis thaliana* (Arabidopsis) Col-0 increased virulence of *P. syringae* pv. *tomato* (Pto) strain DC3000 *hopk1^-^* (Halane et al., 2018). Furthermore, AvrRps4^N^ was necessary and sufficient to trigger a strong hypersensitive response (HR) in resistant lettuce cultivars (Halane et al., 2018; Su et al., 2021). When co-expressed in resistant lettuce with AvrRps4^C^, this AvrRps4^N^-triggered HR phenotype was reduced. In contrast, full avirulence in Arabidopsis Col-0 requires the presence of both AvrRps4^N^ and AvrRps4^C^ (Halane et al., 2018). These dual roles of AvrRps4^N^ are the hallmarks of an effector: benefiting the pathogen while being detrimental enough to some plants that they have evolved to detect it. As for a virulence function, reports directly examining the N-terminus suggest AvrRps4^N^ interacts with EDS1 (Bhattacharjee et al., 2011; Halane et al., 2018), an important immune regulator for TNL proteins and basal resistance (Dongus and Parker, 2021). Interaction with EDS1 has also been reported for the unrelated *P. syringae* pv. *glycinea* effector AvrA1 when transiently expressed in *N. benthamiana* (Wang et al., 2014). For both AvrRps4^N^ and AvrA1 the immediate functional effects of their interactions with EDS1 in terms of virulence or avirulence have not been determined. Taken together, these works highlight a need for greater understanding of AvrRps4^N^ functions.

Contrastingly, AvrRps4^C^ functions are well understood. An electronegative patch (Sohn et al., 2012) interacts with defense-related WRKY transcription factors (Kim et al., 2024) and with the C-terminal integrated WRKY decoy domain of resistance protein RRS1 (Sarris et al., 2015; Mukhi et al., 2021; Kim et al., 2024), providing a mechanism for earlier observations of AvrRps4-dependent suppression of HR in tobacco (Fujikawa et al., 2006; Cvetkovska and Vanlerberghe, 2012), pattern-triggered immunity (PTI) in Arabidopsis, and enhancement of pathogen growth within *N. benthamiana* (Sohn et al., 2009). Processing of AvrRps4 is not necessary for AvrRps4^C^-specific triggering of HR in cultivars of *Brassica rapa* (turnip) nor for resistance to be triggered in Arabidopsis, but is necessary for its virulence function (Sohn et al., 2009). Other work has shown that AvrRps4^C^ directly interacts with BRUTUS (BTS), an iron-sensing protein in the nucleus, causing interference with the degradation of bHLH115 and ILR3 and, when not recognized, leading to increased levels of apoplastic iron (Xing et al., 2021).

## XopO

XopO was first described in 2004 from *Xanthomonas euvesicatoria* pv. *vesicatoria* (Xev) str. 85-10 and is only known to exist in this strain (formerly: *Xanthomonas axonopodis* pv. *vesicatoria* and *Xanthomonas campestris* pv. *vesicatoria*) and *Xanthomonas oryzae* pv. *oryzicola* (Xoc), neither of which appear to be greatly affected by its deletion (Roden et al., 2004; Hajri et al., 2009, 2012; Liao et al., 2020). Indeed, in multiple strains XopO was found to be mutationally inactivated or to possess insertions or deletions within the first 100 amino acids of the protein, suggesting an evolutionary advantage in removing the effector from the secretion repertoire (Wang et al., 2011, p. 20; Barak et al., 2016).

It is not known whether XopO^C^ interacts with WRKY proteins in the same manner as AvrRps4^C^, but we do know that XopO^C^ does not trigger HR in turnip as AvrRps4^C^ does and likewise does not appear to weaken the recognition of XopO^N^ in lettuce to the same degree that AvrRps4^C^ does for AvrRps4^N^ (Sohn et al., 2009; Nguyen et al., 2022). These amino acid polymorphisms and the differential recognition between AvrRps4^C^ and XopO^C^ may be related to functionality within specific plant hosts, and future functional analyses of these effectors should take this into consideration.

## HopK1

HopK1 (previously HopPtoK) was identified in 2002 from Pto DC3000 (Petnicki-Ocwieja et al., 2002). HopK1 in its full length is found almost exclusively within pathovars of *P. syringae*, although not every pathovar or strain carries it. After AvrRps4, HopK1 is the most well-researched bacterial effector protein in the AvrRps4 family.

In Pto DC3000, the *hopK1* gene is located on the chromosome and is encoded in a 24 kb Tn*6022*-like element, but in other strains *hopK1* may occur on a plasmid (Li et al., 2014; Peters et al., 2014). In the case of Pto DAPP-PG 215, *hopK1* is borne on p107, a small plasmid which possesses two prophage regions and some precursors for coronatine production in addition to the effector (Orfei et al., 2023).

Pto DC3000 *hopk1^-^* strains are significantly reduced in their ability to grow and cause disease within plants. The full wild-type growth of the mutant in Arabidopsis Col-0 was not restored by simultaneous transgenic expression of HopK1^C^ but was restored by expression in the same manner of the full-length protein. The N-terminus was also necessary for reduction in reactive oxygen species (ROS) and callose deposition, both indicators of PTI, indicating that HopK1^N^ is indispensable for effector virulence activities (Li et al., 2014). Presence of HopK1 in an otherwise effectorless Pto DC3000 strain did not contribute to nor inhibit growth compared to Pto DC3000 with no effectors at all, nor did it reduce production of ROS in *N. benthamiana.* Nevertheless, authors reported a moderate reduction in “non-avirulence-related” cell death caused by other effectors when HopK1 was present (Wei et al., 2018). Finally, expression of HopK1 did not suppress flg22-mediated activation of FRK1-LUC, another PTI indicator, in Arabidopsis protoplasts (He et al., 2006). Together, these findings may indicate that HopK1^N^ works synergistically with an unknown Pto DC3000 effector(s) in order to counteract early PTI events. This synergistic interaction resulting in reduced ROS and callose may also be required for maximum effectiveness of HopK1, as HopK1^C^ has been shown to travel intercellularly up to one cell layer away by using plasmodesmata as long as movement is not restricted by excess callose deposition (Li et al., 2021; Iswanto et al., 2022).

When infiltrated into Arabidopsis Col-0 alongside AvrRps4^C^, HopK1^N^ fully complements the resistance response seen when AvrRps4^N^ and AvrRps4^C^ are infiltrated together. The central conserved portion of the N-terminus containing the positively charged R112 is required for recognition in lettuce (Halane et al., 2018; Su et al., 2021; Nguyen et al., 2022). However, processing is not required for full pathogenicity (Li et al., 2014).

As mentioned above with AvrRps4, HopK1 was bioinformatically predicted to localize to the chloroplast and was shown to localize to the chloroplast stroma when inducibly expressed by transgenic Arabidopsis, yet nucleo-cytoplasmically when expressed by *A. tumefaciens* in a constitutive transient manner in *N. benthamiana* (Li et al., 2014; De Torres Zabala et al., 2015).

## XopAK

Perhaps representative of its presence in many *Xanthomonas* species with different life histories, *xopAK* is variable in length and number of accumulated mutations. For example, the gene showed very few polymorphic sites among strains of *X. axonopodis manihotis* (Bart et al., 2012; Trujillo et al., 2014) but is believed to be inactivated in *X. phaseoli* pv. *dieffenbachiae* due to a frameshift (Constantin et al., 2017) and is in some cases missing a significant coding region that nearly halves the length of the protein (Fan et al., 2022). Machine learning predicted the presence of a deaminase catalytic domain at the C-terminus of XopAK and at least one potential myristoylation or palmitoylation motif which could target the effector to the plasma membrane (Teper et al., 2015; Barak et al., 2016). This putative deaminase domain is well within the region of homology with HopK1^C^ and may therefore contribute to a more thorough picture of the AvrRps4 family’s *in planta* functions.

Like HopK1, XopAK had no significant effect on flg22-triggered activation of FRK1 in Arabidopsis protoplasts (He et al., 2006; Popov et al., 2016). Unlike *hopK1*, deletion of *xopAK* from Xoc caused no visible changes in disease symptoms on its host (rice) (Liao et al., 2020).

## Discussion

The AvrRps4-type N-terminus is a metaphorical black box for researchers of this family. Its proposed functional region is very small, only about 37 aa long (Su et al., 2021), yet it provokes a strong HR from lettuce (Halane et al., 2018; Su et al., 2021), interacts with a major TNL resistance hub (EDS1) (Bhattacharjee et al., 2011; Halane et al., 2018), and appears more important for the growth of Pto DC3000 than HopK1^C^ alone (Li et al., 2014; Halane et al., 2018), but we still know almost nothing about its function. Intriguingly, using AvrRps4^N^ similarly to pEDV for delivery of the full-length unrelated effector XopQ into *N. benthamiana* cells resulted in a weaker response by the plant than when XopQ was delivered by the AvrRpt2 signal peptide (Gantner et al., 2018). Authors did not test further but hypothesized that this weakness could be the result of differences in stability or translocated amount of protein. While this may be the case, it is also possible that AvrRps4^N^ interaction with EDS1 interferes with the EDS1-mediated recognition of XopQ (Adlung et al., 2016), but more study is needed.

We also do not yet know how detrimental simply possessing an endogenous *avrRps4^N^* might be for bacteria themselves. *xopO* and *xopAK* both naturally occur within the genome of Xev 85-10, but the same is not true for *avrRps4* and *hopK1*, which to date have never been found to naturally co-occur. There are no examples of a strain possessing multiple members of the AvrRps4 effector family (*sensu stricto*), multiple copies of a single member, the *avrRps4*-type C-terminus without an attached N-terminus, or even a single copy of the *avrRps4*-type N-terminus without an attached C-terminus of any type. It is tempting to conclude from this that AvrRps4^N^ may be detrimental to the bacterium over evolutionary time, and we look forward to any future work examining AvrRps4^N^ more thoroughly.

Neither HopK1^C^ nor XopAK cause any significant effect on flg22-triggered FRK1 activation (He et al., 2006; Popov et al., 2016), an early PTI response, yet HopK1 has still been shown to block or reduce HR and PTI (Jamir et al., 2004; Li et al., 2014; Gimenez-Ibanez et al., 2018; Wei et al., 2018). This reduction appears to be related to the action of HopK1^N^ and to be essential for the full virulence of HopK1^C^, yet its homologue XopAK occurs in many more species of *Xanthomonas* without the N-terminus-bearing XopO than it does with it. In fact, their co-occurrence is limited to the only two known pathovars possessing XopO: Xev and Xoc. This raises the question whether XopAK benefits from another effector with redundant function when XopO is not present, or if polymorphisms between the homologues render them dissimilar enough that a ‘helper’ effector is not necessary for XopAK. In the latter case, addition of XopO may have presented an opportunity for range expansion by the effector and the pathogen alike.

As summarized in this review, the presence of *xopO* and *xopAK* within the same strains of *Xanthomonas*, *hopK1* association with transposable elements (Peters et al., 2014; Orfei et al., 2023), and *avrRps4* and *hopK1*’s occasional location on plasmids (Kim et al., 1998; Chang et al., 2005; Orfei et al., 2023) seem to indicate that our modern chimeric effectors may have been formed in a *Xanthomonas* melting pot and then shared with bacteria inhabiting a similar niche, such as *Pseudomonas*, via horizontal gene transfer (HGT). HGT has been implicated in the evolution of *Pseudomonas* (Kim et al., 1998; Rohmer et al., 2004; Dillon et al., 2019) and *Xanthomonas* (Merda et al., 2017; Ruh et al., 2017; Chen et al., 2018) pathogenicity in the past, though not specifically for the AvrRps4 effector family. The rapidity and therefore the agricultural implications of these transfers could be uncovered with examination of the evolutionary history of the family.

As the 30^th^ anniversary of the discovery and cloning of *avrRps4* approaches, we hope investigators will revisit the last 3 decades of AvrRps4 research and open questions with renewed interest, paving the way for (at least)! 3 more decades of insightful molecular plant-pathogen interactions research.

## Author contributions

KH: Conceptualization, Data curation, Writing – original draft, Writing – review & editing. WG: Conceptualization, Funding acquisition, Supervision, Writing – review & editing.
